# What’s This? Another “Consumption Cure”?

**Published:** 1896-03

**Authors:** 


### WHAT'S THIS? ANOTHER "CONSUMPTION CURE"?

It may have been noticed that "consumption cures" appear periodically. The proper (?) method of procedure now seems to be, first to "discover" the "cure," next to describe it fully in some medical journal, with the details of its manufacture and its composition made into a Chinese puzzle, next to sell it to a "Chemical Company," and to get it well ventilated in the daily papers. It may then be seen among the advertising pages of some medical journal, with some such pleasing clause as "None but physicians can use it."

The latest discovery, or rather invention, of this character, is announced by one Dr. Cyrus Edson, of New York, late Health Commissioner of that city, and medical expert on various proprietory compounds, rejuvenators, brain coolers, food assimilators, microbe destroyers, etc. The *Medical Record*, of February 8, contained the official announcement by Dr. Edson, under the title "A Rational Treatment for Phthisis Pulmonalis, together with Some Notes of a New Remedial Solution." Of course it was a mere coincidence that the New York *World*, of same date, contained a chance interview with the "discoverer" upon the same subject. We have as yet seen no explanation of the fact that stacks of the *Record* containing this article were offered for sale on public news-stands, while red pasters announced that Dr. Edson's "Cure" was described therein. The name of this great cure is *Aseptolin.* We can but admire the broad philanthropy of the author when he tells us that "The moment I was assured that my experiment was a success I decided to give the results to the entire profession. It is everybody's property, and it may be used in hospitals and anywhere else." He then proceeds to say that "Aseptolin" is simply a mixture of phenol and pilocarpine-phenyl-hydroxide in water. Now we are told that this pilocarpine-phenyl-hydroxide is a "colorless crystalline salt, new to the medical profession," although it is just a combination of pilocarpine with phenol, from which "all the impurities which have hitherto bothered the profession have been eliminated." So that to produce this remarkable "Aseptolin," we have only to take:

| | |
|---|---|
| Water ($H_2O$) | 97.2411 |
| Phenol ($C_6H_7O$) | 2.7401 |
| Pilocarpine-phenyl-hydroxide ($C_{11}H_{16}N_2O_2OHC_6H_5$) | 0.0188 |

*Quod erat faciendum.* And there you have it. Could anything be simpler? However, Dr. Edson says that it requires extreme care to make, and that his laboratory assistant could not succeed in producing a satisfactory fluid oftener than once in three times. The dose (hypodermic) is about seventy minims to begin with, increasing ten minims per day until one hundred are reached, which is then retained daily "until the patient recovers." But the virtues of pilocarpine-phenyl-hydroxide are not limited exclusively to the cure of phthisis, though one would suppose that would be honor enough. "My experience with pilocarpine-phenyl-hydroxide in the treatment of *malaria** leads me to consider it a specific, and of even greater efficiency than quinine." Also useful in "*catarrhal and febricular grippe.**" "I confidently believe this method of treatment will afford the best result yet obtained, not only in the cure of phthisis and other forms of tuberculosis, but of other diseases of germ origin. The possible curative range of the fluid is obviously very wide."

The next chapter in this book of discovery may be found in the daily papers, and with this we close:

TO PHYSICIANS IN REGULAR PRACTICE

Will be sent free by mail a sample bottle of Dr. Edson's Aseptolin, the newly discovered treatment for consumption, etc., together with Dr. Edson's paper, reprinted from the *N. Y. Medical Record*, of February 8, 1896. Those who have patients suffering from consumption are urged to test this remedy. None but physicians can use it. Address ——— Chemical Co., ——— St., New York."

